# Different level of population differentiation among human genes

**DOI:** 10.1186/1471-2148-11-16

**Published:** 2011-01-14

**Authors:** Dong-Dong Wu, Ya-Ping Zhang

**Affiliations:** 1 State Key Laboratory of Genetic Resources and Evolution, Kunming Institute of Zoology, Chinese Academy of Sciences, Kunming, PR China; 2 Laboratory for Conservation and Utilization of Bio-resource, Yunnan University, Kunming 650091, PR China; 3 Graduate School of the Chinese Academy of Sciences, Beijing, PR China

## Abstract

**Background:**

During the colonization of the world, after dispersal out of African, modern humans encountered changeable environments and substantial phenotypic variations that involve diverse behaviors, lifestyles and cultures, were generated among the different modern human populations.

**Results:**

Here, we study the level of population differentiation among different populations of human genes. Intriguingly, genes involved in osteoblast development were identified as being enriched with higher *F*_ST _SNPs, a result consistent with the proposed role of the skeletal system in accounting for variation among human populations. Genes involved in the development of hair follicles, where hair is produced, were also found to have higher levels of population differentiation, consistent with hair morphology being a distinctive trait among human populations. Other genes that showed higher levels of population differentiation include those involved in pigmentation, spermatid, nervous system and organ development, and some metabolic pathways, but few involved with the immune system. Disease-related genes demonstrate excessive SNPs with lower levels of population differentiation, probably due to purifying selection. Surprisingly, we find that Mendelian-disease genes appear to have a significant excessive of SNPs with high levels of population differentiation, possibly because the incidence and susceptibility of these diseases show differences among populations. As expected, microRNA regulated genes show lower levels of population differentiation due to purifying selection.

**Conclusion:**

Our analysis demonstrates different level of population differentiation among human populations for different gene groups.

## Background

After dispersal from Africa, humans have evolved to be characterized by substantial phenotypic variation, including variation in skin, hair, and eye color, body mass, height, diet, drug metabolism, susceptibility and resistance to disease, during the colonization of the World. Efforts to reveal the genetic bases of these variations should provide important insight into the history of human evolution, gene function, and the mechanisms of disease [1,2]. Indeed, with the advent of large scale comparative genomic and human polymorphism data, a flood of studies have identified many candidate genes and genomic regions accounting for the observed phenotypic characters [2]. However, the evolutionary forces, i.e., positive selection, balancing selection, purifying selection, or neutral evolution, driving the variation of these phenotypic traits remain largely unknown.

In general, population differentiation under neutral evolution is mostly influenced by demographic history; however, adaptation to a local environment, driven by positive selection, will increase the level of population differentiation [3]. In contrast, negative and balancing selection tends to reduce population differentiation [3]. Accordingly, the evaluation of the level of population differentiation of the human genome would be helpful and informative for the identification of the genetic basis of the phenotypic difference observed in different human populations.

## Results and Discussion

Here, we evaluated the level of population differentiation for human genes on autosomal chromosomes among three populations: African, European and East Asian, based on the HapMap data (Phase II) [4], using the parameter *F*_ST _according to methods described previously [3,5]. A previous study has reported that there is a higher level of population differentiation at gene regions compared to non-gene regions in the genome [6]. However, in our analysis, we observed that for several chromosomes, including 5, 6, 8, 11, 13, and 20, did not show a pattern with higher population differentiation at genic compared to non-genic regions, namely genic regions did not have excess SNPs with a higher *F*_ST _(≥0.6) (Figure S1 in Additional file 1).

### Functional significance of genes with higher levels of population differentiation

**Figure 1:**
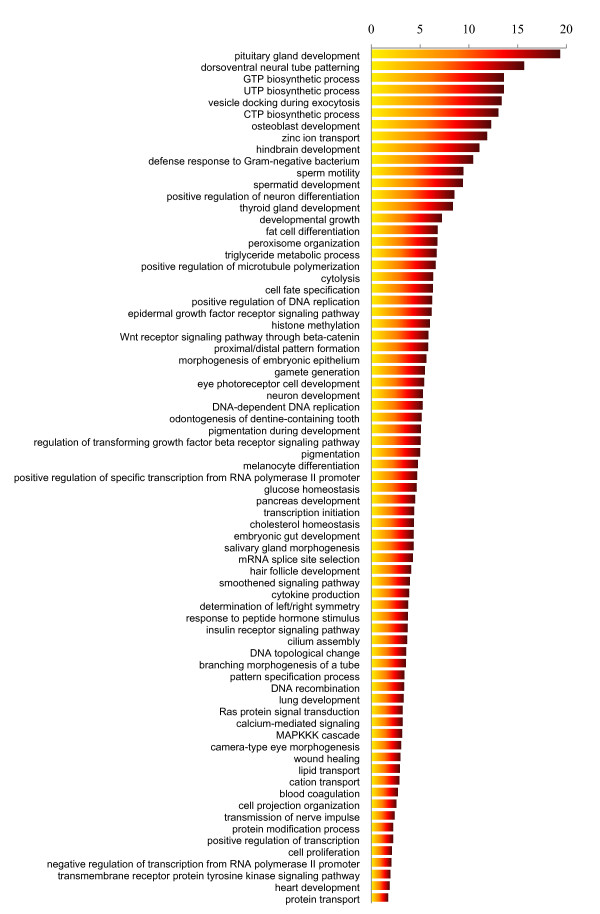
**λ values of GO categories in biological processes enriched for higher *F*_ST _SNPs with *P*-value lower than 10^-10^**.

Since an analysis of categories that contain only a few genes will have low statistical power, here we only summarize categories that contain at least 10 genes. Figure 1 summarizes the biological processes that are enriched with higher *F*_ST _SNPs with a significant *P *value of 10^-10 ^or lower (see Method), and their λ values, with λ being the ratio of the proportion of higher *F*_ST _SNPs (≥0.6) in the analyzed category to the proportion of higher *F*_ST _SNPs in genome-wide genes (which is 0.0049). The categories listed in Figure 1 include a large number involved with organ development, such as those involved in pancreas, lung, and heart development. For example, GO: 0021983, pituitary gland development, is enriched with high *F*_ST _SNPs and has the highest λ value, 19.37. The pituitary gland produces and secretes many hormones, some of which stimulate other glands to produce other types of hormones, thus this organ and it controls many biochemical processes, e.g. growth, homeostasis, stress response, reproduction, and metabolism [7,8], that similarly demonstrate a high level of population differentiation, such as developmental growth (GO: 0048589), reproduction related(GO:0030317, GO:0007286, GO:0007276), and several metabolic pathways (GO: 0006641,GO: 0042593, GO: 0042632) (see following text and Figure 1).

An intriguing observation is that osteoblast development is significantly rich in high *F*_ST _SNPs (λ = 12.28, *P*= 4.92E-88 after multiple testing). Osteoblasts are mononucleate cells that are responsible for bone formation. Modern humans demonstrate substantial phenotypic variation, which to a large extent can be illuminated by the skeletal system, such as height, body mass, body mineral density, and craniofacial differences. Indeed, evidence indicates that the human skeletal system has evolved rapidly since the advent of agriculture [9] and our recent study concluded that the high levels of population differentiation of skeletal genes among human populations was driven by positive selection [10].

Another interesting category is hair follicle development, which also showed a higher level of population differentiation (GO: 0001942, λ = 4.09, *P*= 2.07E-08 after multiple testing). Hair is produced by hair follicles. Similar to the skeletal system, hair morphology, including water swelling diameter and section, shape of fiber, mechanical properties, combability and hair moisture, have distinctive traits among human populations [11]. Previous studies have identified some genes involved in hair follicle development that have undergone recent positive selection, as detected by the long range haplotype homozygosity test, such as *EDAR *and *EDA2R *[12,13]. These studies, together with our evidence of higher population differentiation in the genes involved in the hair follicle development support a hypothesis of adaptive evolution accounting for the diversification of human hair.

Consistent with previous observations [12,14], genes involved in pigmentation, including the following GO processes: pigmentation during development, pigmentation, and melanocyte differentiation, demonstrated significantly higher population differentiation. In a similar manner, reproduction associated processes, e.g. sperm motility, spermatid development, gamete generation, have higher levels of population differentiation (Figure 1). Among the categories with a significant enrichment of higher *F*_ST _SNPs, many are involved in the nervous system, e.g. dorsoventral neural tube patterning (GO: 0021904, λ = 15.67), hindbrain development (GO: 0030902, λ = 11.08), positive regulation of neuron differentiation (GO: 0045666, λ = 8.50), and neuron development (GO: 0048666, λ = 5.27) (Figure 1). Others categories include metabolic process, such as the triglyceride metabolic process (GO: 0006641, λ = 6.69), glucose homeostasis (GO: 0042593, λ = 4.64), cholesterol homeostasis (GO: 0042632, λ = 4.35), possibly resulting from the variation in metabolism among humans.

Immunity-related genes, however, which are a common target of positive selection [2,15,16], are involved in small list of categories with a higher proportion of higher *F*_ST _SNPs. This observation is probably attributable to the fact that many of the genes in the immunity system evolve under balancing selection in human populations for a heterozygote advantage, which would reduce the level of population differentiation [17,18].

Tables S1 in Additional file 2, and Tables S2 in Additional file 3 summarize the GO categories in cellular component and molecular function with an enrichment of higher *F*_ST _SNPs.

**Figure 2:**
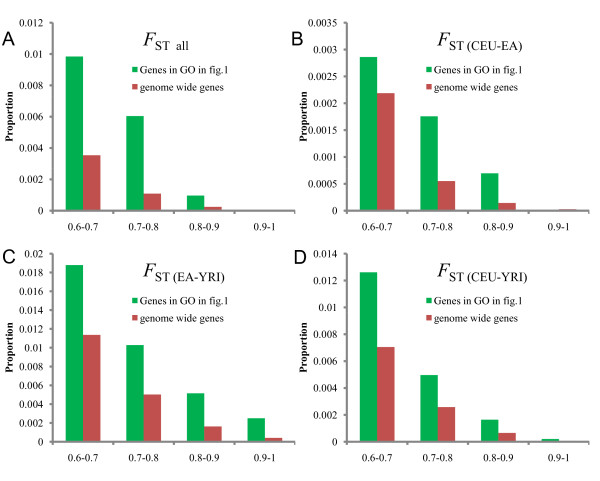
**The *F*_ST _(≥0.6) distribution of SNPs in the biological processes in Figure 1 and of genome-wide genes**. (A) *F*_ST all _-values among the three populations. (B) *F*_ST(CEU-EA) _-values between Europeans and East Asians. (C) *F*_ST(EA-YRI) _-values between East Asians and Africans. (D) *F*_ST(CEU-YRI) _-values between Europeans and Africans.

In addition, to discern which population(s) contribute more to the pattern, we generated three pairwise sets of *F*_ST_-values: *F*_ST (CEU-YRI)_, *F*_ST (EA-YRI) _and *F*_ST (CEU-EA)_. At the genes in the biological processes described in Figure 1, the three data sets demonstrate consistent pattern of significantly higher proportion of higher *F*_ST _SNPs compared with that at the genome-wide genes (Figure 2), which suggested that the population differentiation is present commonly between pairwise populations.

Population differentiation under neutral evolution is mostly influenced by demographic history (that is, genetic drift and gene flow), which can generate similar pattern with biological factor such as natural selection. However, demographic history tends to influence all loci in the genome equally, and natural selection acts only on the single gene or a group of functional related genes. Compared with the proportion of higher *F*_ST _SNPs in the genome-wide genes, we present some groups of functional related genes enriched with high *F*_ST _SNPs, which are mostly driven by positive natural selection, although the confounding factor of demographic history cannot be excluded absolutely.

### Population differentiation in disease-related genes

**Figure 3:**
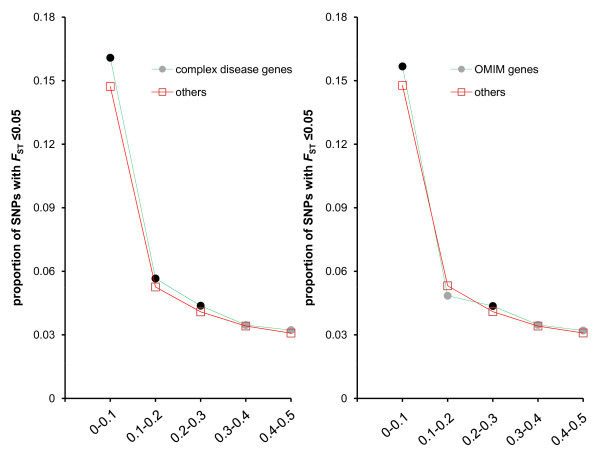
**Proportions of SNPs with *F*_ST _≤ 0.05 at each global MAF (minor allele frequencies) bin in complex disease genes (A), and OMIM genes (B), compared to that of other genes**. The black nodes indicate significantly higher proportion in disease genes with P < 0.01.

Studies of the pattern of molecular evolution of human disease-related genes will provide insight into the origin, maintenance and mechanism of disease [19]. Previous reports suggested that disease-related genes tend to evolve under purifying selection based on the comparison of non-synonymous rate to synonymous substitution rates [19-21]. Here, as expected, we found that disease-related genes (including Mendelian disease genes and complex disease genes), demonstrate a significant excess of SNPs with lower *F*_ST _(≤0.05), relative to other genes (χ^2^= 23.16, *P*= 1.49E-06 for OMIM gene panel, χ^2^= 193.78, *P *= 4.76E-44 for complex-disease gene panel, Figure S2 in Additional file 1). These disease genes demonstrate an excess of lower *F*_ST _SNPs in the lower frequency bins but not in the high frequency bins (Figure 3), suggesting that negative selection, rather than balancing selection, operated on these genes.

**Figure 4:**
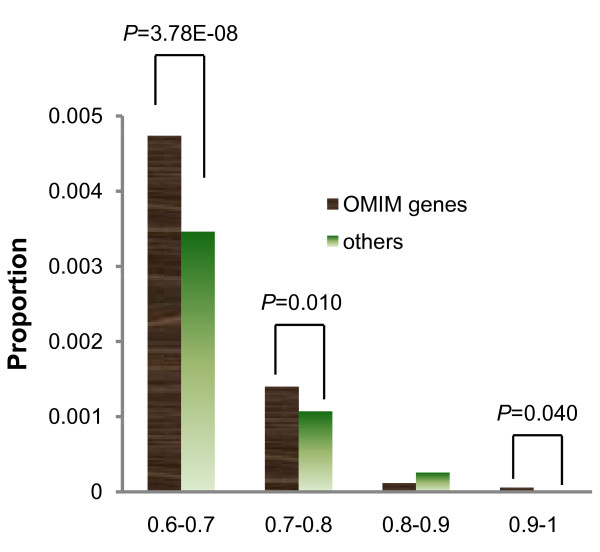
**Proportions of SNPs with *F*_ST _≥ 0.60 at each global MAF (minor allele frequencies) bin in OMIM genes and non-OMIM genes**. The *P*-value with statistical significance is presented above each bin.

Surprisingly, higher *F*_ST _(≥0.6) SNPs are enriched significantly at Mendelian disease genes (OMIM) relative to other genes (χ^2 ^= 30.47, *P *= 3.39E-08), with three MAF bins demonstrating statistical significance (Figure 4). These higher *F*_ST _SNPs are probably under positive selection. This pattern, however, was not observed in complex disease genes and appear inconsistent with the previous study by Blekhman et al. (2008) [20]. Blekhman et al. (2008) found that Mendelian-disease genes appear to be under widespread purifying selection but that genes that influence complex disease risk show lower levels of evolutionary conservation, as assessed by the ratio of nonsynonymous to synonymous substitutions (Dn/Ds), possibly because they were targeted by both purifying and positive selection. The difference in results is probably attributable to the different methods used to assess sequence evolution: Dn/Ds method changes over a long time scale (i.e. between human and other species), while *F*_ST _measures recent evolution (i.e., since the separation of modern human populations). The incidence and susceptibility to some Mendelian diseases might demonstrate higher levels of differences among modern human populations.

### Lower levels of population differentiation in microRNA targeted genes

**Figure 5:**
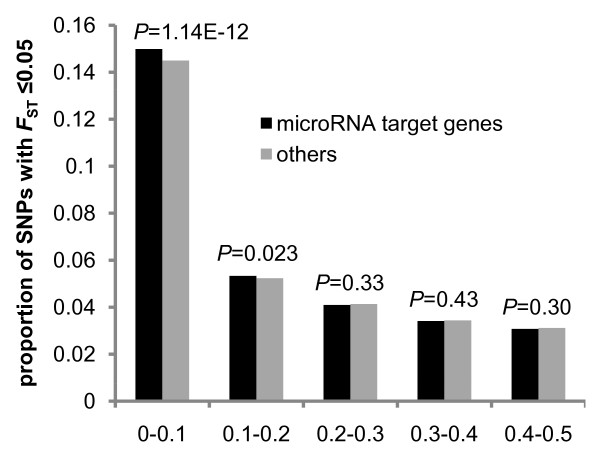
**Proportions of SNPs with *F*_ST _≤ 0.05 at each global MAF (minor allele frequencies) bin for microRNA targeted genes compared with other genes**. The *P*-value is presented above each bin.

The regulation of gene expression is crucial to the development of an organism and has been increasingly recognized that a remarkable fraction of regulation is dominated by microRNAs (miRNAs) [22,23]. miRNAs are a group of ~23 nt endogenous RNAs important for a diverse range of biological functions that direct the posttranscriptional repression of mRNAs by cleavage or translational repression [22,23]. Evidence has shown that negative selection operates on miRNA regulated genes [24]. Here, we observed that microRNA targeted genes present a significant excess of lower *F*_ST _(≤0.05) SNPs (χ^2 ^= 29.76, *P *= 4.90E-08), and significantly fewer high *F*_ST _(≥0.6) SNPs (χ^2 ^= 37.61, *P *= 8.63E-10), relative to other genes (Figure S3 in Additional file 1). The lower *F*_ST _SNPs are mainly restricted within the lower minor allele frequency bins, and not the intermediate frequency bin (Figure 5), suggesting that widespread purifying selection operated on miRNA targeted genes.

## Conclusions

In this study, we find that genes involved in osteoblast development, hair follicles development, pigmentation, spermatid, nervous system and organ development, and some metabolic pathways have higher levels of population differentiation. Surprisingly, we find that Mendelian-disease genes appear to have a significant excessive of SNPs with high levels of population differentiation, possibly because the incidence and susceptibility of these diseases show differences among populations. As expected, microRNA regulated genes show lower levels of population differentiation due to purifying selection. Our analysis demonstrates different level of population differentiation among human populations for different gene groups.

## Methods

Since genes on the sex chromosomes are involved in higher population differentiation than those on the autosomal chromosomes [3], we only analyzed data from the autosomal chromosomes. Allele frequency data for SNPs on autosomes were retrieved from HapMap Phase II (release 24, NCBI36) [4] for three populations: African (YRI panel including 60 Yoruban individuals from Ibadan), European (CEU panel including 60 individuals of Utah residents with ancestry from northern and western Europe) and East Asian (EA panels including 45 Han Chinese (HCB) and 45 Japanese from Tokyo (JPT)).To evaluate the degree of population differentiation, *F*_ST _values of the polymorphic SNPs with minor allele frequencies ≥0.01 in at least one population were calculated as previously described [3,5]. Since negative values have no biological explanation these were set to 0.

Protein coding genes on the human autosomal chromosomes, and their corresponding gene ontology (GO) terms including three categories: biological process, cellular component, and molecular function, were downloaded from Ensembl (http://www.ensembl.org version 54) by means of BioMart [25]. Each gene was extended 500 bp upstream of 5'-termus and downstream of 3'-termus to include all of its SNPs. χ^2 ^tests with one degree of freedom were used to test for the significance of the enrichment of SNPs with higher (≥0.6) *F*_ST _values compared with genome-wide genes empirical data based on 2 × 2 contingency tables constructed by the numbers of SNPs. For these analyses, Bonferroni correction was used for the multiple testing. To better understand the enrichment, we calculated the parameter, λ, the ratio of the proportion of higher *F*_ST _SNPs in the analyzed category to that in the genome-wide genes. λ values significantly higher than 1 indicates a higher population differentiation of genes in the category among human populations.

Complex disease genes were obtained from the Genetic Association Database (GAD) [26]. Human Mendelian disease genes were obtained from the study by Blekhman et al. (2008) (OMIM) [20]. Genes targeted by microRNA were obtained from targetscan (http://www.targetscan.org, release 5.1) [27-29]. For these genes, χ^2 ^tests with one degree of freedom were used to test the significance of an enrichment of SNPs with higher (≥0.6) *F*_ST _values and lower (≤0.05) *F*_ST _values, respectively, compared with other genes based on 2 × 2 contingency tables constructed by the numbers of SNPs.

## Authors' contributions

DDW performed the analyses, analyzed and interpreted the data; DDW and YPZ conceived the study and wrote the paper. All authors read and approved the final manuscript.

## Supplementary Material

Additional file 1 W**ord file including Figure S1, FigureS2 and Figure S3**. Click here for file

Additional file 2 W**ord file including Table S1**. Click here for file

Additional file 3 W**ord file including Table S2**. Click here for file

## References

[B1] Novembre J Di Rienzo A Spatial patterns of variation due to natural selection in humans Nat Rev Genet 2009 10 11 745 755 10.1038/nrg2632 19823195 PMC3989104

[B2] Sabeti PC Schaffner SF Fry B Lohmueller J Varilly P Shamovsky O Palma A Mikkelsen TS Altshuler D Lander ES Positive natural selection in the human lineage Science 2006 312 5780 1614 1620 10.1126/science.1124309 16778047

[B3] Akey JM Zhang G Zhang K Jin L Shriver MD Interrogating a high-density SNP map for signatures of natural selection Genome Res 2002 12 12 1805 1814 10.1101/gr.631202 12466284 PMC187574

[B4] The International HapMap Consortium A second generation human haplotype map of over 3.1 million SNPs Nature 2007 449 851 861 10.1038/nature06258 17943122 PMC2689609

[B5] Weir BS Cockerham CC Estimating F-statistics for the analysis of population structure Evolution 1984 38 1358 1370 10.2307/2408641 28563791

[B6] Barreiro LB Laval G Quach H Patin E Quintana-Murci L Natural selection has driven population differentiation in modern humans Nat Genet 2008 40 3 340 345 10.1038/ng.78 18246066

[B7] Hojo M Kita A Kageyama R Hashimoto N Notch-Hes signaling in pituitary development Expert Review of Endocrinology & Metabolism 2008 3 1 91 100 10.1586/17446651.3.1.91 30743788

[B8] Zhu X Gleiberman AS Rosenfeld MG Molecular physiology of pituitary development: signaling and transcriptional networks Physiol Rev 2007 87 3 933 963 10.1152/physrev.00006.2006 17615393

[B9] Larsen CS Biological changes in human populations with agriculture Ann Rev Anthropol 1995 24 1 185 213 10.1146/annurev.an.24.100195.001153

[B10] Wu DD Zhang YP Positive selection drives population differentiation in the skeletal genes in modern humans Hum Mol Genet 2010 19 2341 2346 10.1093/hmg/ddq107 20233747

[B11] Franbourg A Hallegot P Baltenneck F Toutain C Leroy F Current research on ethnic hair J Am Acad Dermatol 2003 48 6PB 115 119 10.1067/mjd.2003.277 12789163

[B12] Sabeti PC Varilly P Fry B Lohmueller J Hostetter E Cotsapas C Xie X Byrne EH McCarroll SA Gaudet R Genome-wide detection and characterization of positive selection in human populations Nature 2007 449 7164 913 918 10.1038/nature06250 17943131 PMC2687721

[B13] Fujimoto A Kimura R Ohashi J Omi K Yuliwulandari R Batubara L Mustofa MS Samakkarn U Settheetham-Ishida W Ishida T A scan for genetic determinants of human hair morphology: EDAR is associated with Asian hair thickness Hum Mol Genet 2008 17 6 835 843 10.1093/hmg/ddm355 18065779

[B14] Izagirre N Garcia I Junquera C de la Rua C Alonso S A scan for signatures of positive selection in candidate loci for skin pigmentation in humans Mol Biol Evol 2006 23 9 1697 1706 10.1093/molbev/msl030 16757656

[B15] Wu DD Zhang YP Positive Darwinian selection in human population: A review Chinese Science Bulletin 2008 53 10 1457 1467 10.1007/s11434-008-0202-z

[B16] Vallender EJ Lahn BT Positive selection on the human genome Hum Mol Genet 2004 13 Review Issue 2 R245 R254 10.1093/hmg/ddh253 15358731

[B17] Ferrer-Admetlla A Bosch E Sikora M Marques-Bonet T Ramirez-Soriano A Muntasell A Navarro A Lazarus R Calafell F Bertranpetit J Balancing selection is the main force shaping the evolution of innate immunity genes J Immunol 2008 181 2 1315 1322 18606686 10.4049/jimmunol.181.2.1315

[B18] Fumagalli M Cagliani R Pozzoli U Riva S Comi GP Menozzi G Bresolin N Sironi M Widespread balancing selection and pathogen-driven selection at blood group antigen genes Genome Res 2009 19 2 199 212 10.1101/gr.082768.108 18997004 PMC2652214

[B19] Cai JJ Borenstein E Chen R Petrov DA Similarly strong purifying selection acts on human disease genes of all evolutionary ages Genome Biol Evol 2009 2009 0 131 144 10.1093/gbe/evp013 PMC2817408 20333184

[B20] Blekhman R Man O Herrmann L Boyko AR Indap A Kosiol C Bustamante CD Teshima KM Przeworski M Natural selection on genes that underlie human disease susceptibility Curr Biol 2008 18 12 883 889 10.1016/j.cub.2008.04.074 18571414 PMC2474766

[B21] Nielsen R Hubisz MJ Hellmann I Torgerson D Andres AM Albrechtsen A Gutenkunst R Adams MD Cargill M Boyko A Darwinian and demographic forces affecting human protein coding genes Genome Res 2009 19 5 838 849 10.1101/gr.088336.108 19279335 PMC2675972

[B22] Bartel DP MicroRNAs genomics, biogenesis, mechanism, and function Cell 2004 116 2 281 297 10.1016/S0092-8674(04)00045-5 14744438

[B23] Bartel DP MicroRNAs: target recognition and regulatory functions Cell 2009 136 2 215 233 10.1016/j.cell.2009.01.002 19167326 PMC3794896

[B24] Chen K Rajewsky N Natural selection on human microRNA binding sites inferred from SNP data Nat Genet 2006 38 12 1452 1456 10.1038/ng1910 17072316

[B25] Smedley D Haider S Ballester B Holland R London D Thorisson G Kasprzyk A BioMart - biological queries made easy BMC Genomics 2009 10 1 22 10.1186/1471-2164-10-22 19144180 PMC2649164

[B26] Becker KG Barnes KC Bright TJ Wang SA The genetic association database Nat Genet 2004 36 5 431 432 10.1038/ng0504-431 15118671

[B27] Grimson A Farh KKH Johnston WK Garrett-Engele P Lim LP Bartel DP MicroRNA targeting specificity in mammals: determinants beyond seed pairing Mol Cell 2007 27 1 91 105 10.1016/j.molcel.2007.06.017 17612493 PMC3800283

[B28] Lewis BP Burge CB Bartel DP Conserved seed pairing, often flanked by adenosines, indicates that thousands of human genes are microRNA targets Cell 2005 120 1 15 20 10.1016/j.cell.2004.12.035 15652477

[B29] Friedman RC Farh KKH Burge CB Bartel DP Most mammalian mRNAs are conserved targets of microRNAs Genome Res 2009 19 1 92 105 10.1101/gr.082701.108 18955434 PMC2612969

