# Supplementary material for: Different level of population differentiation among human genes
Source: BMC Evol Biol. 2011 Jan 14;11:16. doi: 10.1186/1471-2148-11-16 (PMC3032687; doi:10.1186/1471-2148-11-16)
Supplement: Additional file 1 — Word file including Figure S1, FigureS2 and Figure S3. [file 1471-2148-11-16-S1.DOC]

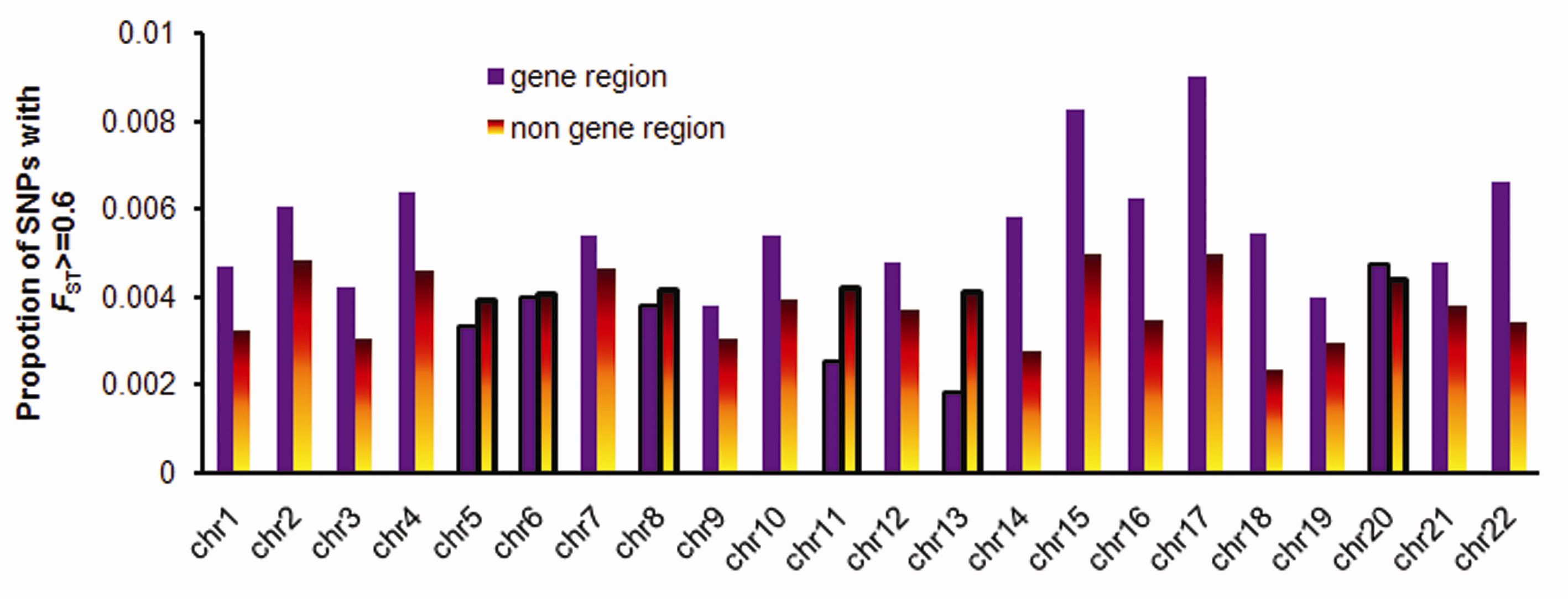


Figure S1: The proportion of SNPs with Fst ≥ 0.6 at the gene region and non gene region in the 22 auto chromosomes. The bolds are chromosomes that do not have the pattern that the proportion of SNPs at the gene region is higher that at non gene region.


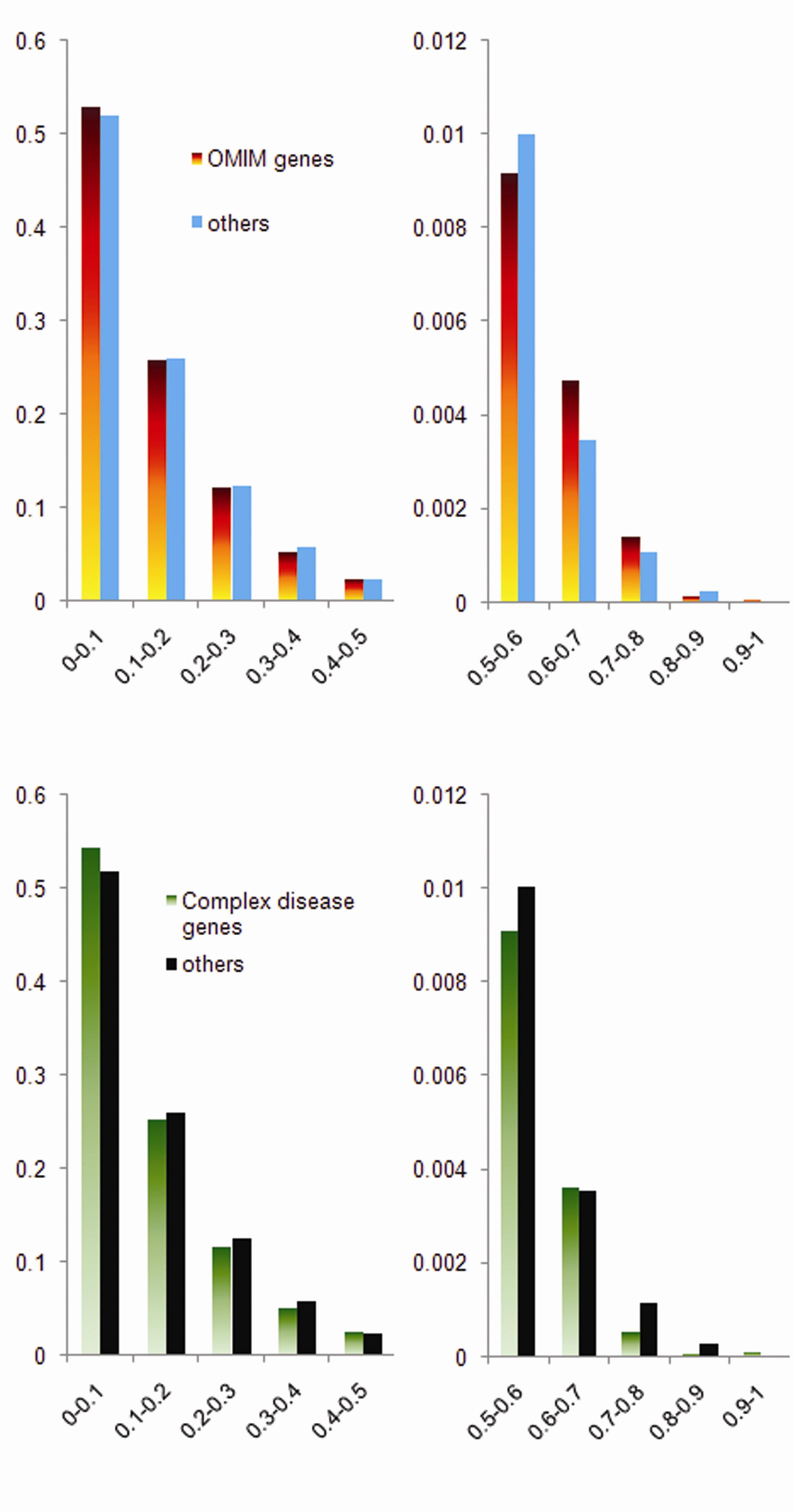


Figure S2: The *F*_ST_ distribution of SNPs in the disease genes and that of other genes


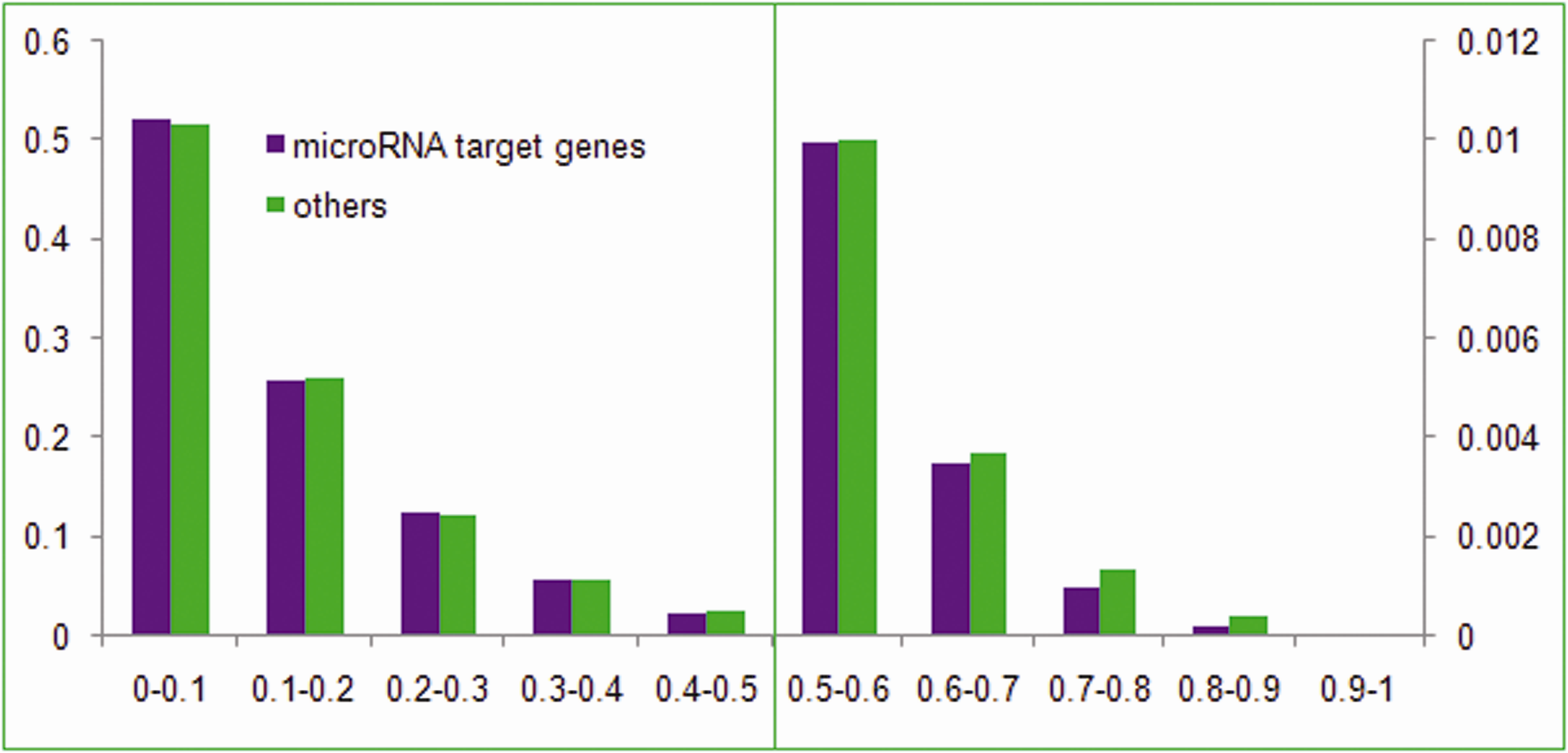


Figure S3: The *F*_ST_ distribution of SNPs in the microRNA target genes and that of other genes.
