# Supplementary material for: Different level of population differentiation among human genes
Source: BMC Evol Biol. 2011 Jan 14;11:16. doi: 10.1186/1471-2148-11-16 (PMC3032687; doi:10.1186/1471-2148-11-16)
Supplement: Additional file 2 — Word file including Table S1. [file 1471-2148-11-16-S2.DOC]

Table S1: GO categories in cellular component with enrichment of higher *F*_ST_ SNPs with P-value lower than 10^-10^.

| GO | GO Description | Gene number | higher Fst SNPs | total SNPs | χ2 | P-value | λ |
| --- | --- | --- | --- | --- | --- | --- | --- |
| GO:0000794 | condensed nuclear chromosome | 20 | 50 | 858 | 497.191 | 3.88E-110 | 11.894 |
| GO:0012506 | vesicle membrane | 11 | 41 | 765 | 369.516 | 2.39E-82 | 10.938 |
| GO:0005680 | anaphase-promoting complex | 14 | 36 | 704 | 306.837 | 1.07E-68 | 10.437 |
| GO:0005740 | mitochondrial envelope | 16 | 18 | 242 | 238.892 | 6.86E-54 | 15.181 |
| GO:0000922 | spindle pole | 24 | 35 | 949 | 197.920 | 5.94E-45 | 7.527 |
| GO:0045121 | membrane raft | 61 | 78 | 4676 | 131.400 | 2.02E-30 | 3.404 |
| GO:0005938 | cell cortex | 40 | 58 | 3328 | 106.105 | 6.99E-25 | 3.557 |
| GO:0005813 | centrosome | 171 | 147 | 13350 | 99.816 | 1.67E-23 | 2.247 |
| GO:0019861 | flagellum | 31 | 45 | 2560 | 83.769 | 5.56E-20 | 3.588 |
| GO:0005819 | spindle | 62 | 63 | 4525 | 74.771 | 5.29E-18 | 2.842 |
| GO:0005667 | transcription factor complex | 141 | 96 | 8397 | 72.342 | 1.81E-17 | 2.333 |
| GO:0009925 | basal plasma membrane | 22 | 21 | 909 | 61.557 | 4.30E-15 | 4.715 |
| GO:0005694 | chromosome | 186 | 80 | 7187 | 56.484 | 5.66E-14 | 2.272 |
| GO:0005929 | cilium | 71 | 96 | 9441 | 52.902 | 3.51E-13 | 2.075 |
| GO:0031093 | platelet alpha granule lumen | 31 | 32 | 1965 | 51.964 | 5.65E-13 | 3.324 |
