# Supplementary material for: Different level of population differentiation among human genes
Source: BMC Evol Biol. 2011 Jan 14;11:16. doi: 10.1186/1471-2148-11-16 (PMC3032687; doi:10.1186/1471-2148-11-16)
Supplement: Additional file 3 — Word file including Table S2. [file 1471-2148-11-16-S3.DOC]

Table S2: GO categories in molecular function with enrichment of higher *F*_ST_ SNPs with P-value lower than 10^-10^.

| GO | GO Description | gene number | higher Fst SNPs | total SNPs | X^2^ | P-value | λ |
| --- | --- | --- | --- | --- | --- | --- | --- |
| GO:0008324 | cation transmembrane transporter activity | 38 | 91 | 2263 | 570.104 | 5.33E-126 | 8.207 |
| GO:0004550 | nucleoside diphosphate kinase activity | 10 | 32 | 481 | 372.698 | 4.84E-83 | 13.578 |
| GO:0031177 | phosphopantetheine binding | 20 | 51 | 1223 | 336.844 | 3.11E-75 | 8.511 |
| GO:0016881 | acid-amino acid ligase activity | 33 | 123 | 5927 | 299.348 | 4.57E-67 | 4.235 |
| GO:0005086 | ARF guanyl-nucleotide exchange factor activity | 16 | 56 | 2228 | 185.370 | 3.26E-42 | 5.130 |
| GO:0019904 | protein domain specific binding | 59 | 55 | 2846 | 120.365 | 5.26E-28 | 3.944 |
| GO:0005003 | ephrin receptor activity | 14 | 52 | 2928 | 98.458 | 3.32E-23 | 3.625 |
| GO:0050839 | cell adhesion molecule binding | 12 | 23 | 841 | 86.592 | 1.33E-20 | 5.582 |
| GO:0004843 | ubiquitin-specific protease activity | 16 | 23 | 853 | 84.843 | 3.23E-20 | 5.503 |
| GO:0003918 | DNA topoisomerase (ATP-hydrolyzing) activity | 17 | 46 | 2770 | 77.267 | 1.49E-18 | 3.389 |
| GO:0003700 | transcription factor activity | 966 | 431 | 57193 | 76.033 | 2.79E-18 | 1.538 |
| GO:0016566 | specific transcriptional repressor activity | 23 | 44 | 2716 | 70.618 | 4.34E-17 | 3.306 |
| GO:0008289 | lipid binding | 113 | 75 | 6059 | 68.642 | 1.18E-16 | 2.526 |
| GO:0016779 | nucleotidyltransferase activity | 60 | 54 | 3951 | 61.737 | 3.93E-15 | 2.789 |
| GO:0004190 | aspartic-type endopeptidase activity | 35 | 21 | 938 | 58.633 | 1.90E-14 | 4.569 |
| GO:0005070 | SH3/SH2 adaptor activity | 43 | 67 | 5626 | 56.063 | 7.02E-14 | 2.431 |
| GO:0015269 | calcium-activated potassium channel activity | 14 | 36 | 2378 | 50.827 | 1.01E-12 | 3.090 |
| GO:0016563 | transcription activator activity | 167 | 128 | 13993 | 50.698 | 1.08E-12 | 1.867 |
| GO:0008144 | drug binding | 17 | 21 | 1070 | 47.427 | 5.71E-12 | 4.006 |
| GO:0000166 | nucleotide binding | 1715 | 884 | 142197 | 44.062 | 3.18E-11 | 1.269 |
| GO:0016301 | kinase activity | 112 | 84 | 8432 | 43.706 | 3.82E-11 | 2.033 |
| GO:0008138 | protein tyrosine/serine/threonine phosphatase activity | 45 | 32 | 2157 | 43.441 | 4.37E-11 | 3.028 |
| GO:0003887 | DNA-directed DNA polymerase activity | 29 | 30 | 1968 | 42.975 | 5.55E-11 | 3.111 |
